# The First Complete Mitochondrial Genome of Genus *Isocapnia* (Plecoptera: Capniidae) and Phylogenetic Assignment of Superfamily Nemouroidea

**DOI:** 10.3390/genes14050965

**Published:** 2023-04-24

**Authors:** Abdur Rehman, Qing-Bo Huo, Yu-Zhou Du

**Affiliations:** 1 College of Plant Protection & Institute of Applied Entomology, Yangzhou University, Yangzhou 225009, China; 2 Joint International Research Laboratory of Agriculture and Agri-Product Safety, The Ministry of Education, Yangzhou University, Yangzhou 225009, China

**Keywords:** Plecoptera, Capniidae, *Isocapnia*, mitogenome, phylogeny

## Abstract

Capniidae are a family of stoneflies, also known as snow flies, who emerge in winter. The phylogeny of Capniidae is widely accepted to be based on morphological analysis. Until now, only five Capniidae mitochondrial genomes have been sequenced so far. In addition, sampling is required to determine an accurate phylogenetic association because the generic classification of this family is still controversial and needs to be investigated further. In this study, the first mitogenome of genus *Isocapnia* was sequenced with a length of 16,200 bp and contained 37 genes, including a control region, two rRNAs, 22 tRNAs, and 13 PCGs, respectively. Twelve PCGs originated with the common start codon ATN (ATG, ATA, or ATT), while *nad5* used GTG. Eleven PCGs had TAN (TAA or TAG) as their last codon; however, *cox1* and *nad5* had T as their final codon due to a shortened termination codon. All tRNA genes demonstrated the cloverleaf structure, which is distinctive for metazoans excluding the *tRNASer1 (AGN)* that missed the dihydrouridine arm. A Phylogenetic analysis of the superfamily Nemouroidea was constructed using thirteen PCGs from 32 formerly sequenced Plecoptera species. The Bayesian inference and maximum likelihood phylogeny tree structures derived similar results across the thirteen PCGs. Our findings strongly supported Leuctridae + ((Capniidae + Taeniopterygidae) + (Nemouridae + Notonemouridae)). Ultimately, the best well-supported generic phylogenetic relationship within Capniidae is as follows; (*Isocapnia* + (*Capnia + Zwicknia*) + (*Apteroperla + Mesocapnia*)). These findings will enable us to better understand the evolutionary relationships within the superfamily Nemouroidea and the generic classification and mitogenome structure of the family Capniidae.

## 1. Introduction

The mitogenome is a fully functional and relatively independent organelle encompassing general information ranging from molecular studies to genetic variants [1]. Mitogenome research is frequently used in taxonomy, phylogenetics, and evolutionary studies in many animals. It has been extensively studied to resolve insect phylogeny [2,3,4]. Metazoans often have circular, double-standard molecules in their mitogenomes. It comprises two ribosomal RNA genes (rRNAs), 22 transfer RNA genes (tRNAs), 13 protein-coding genes (PCGs), and a non-coding region (CR) [5,6,7].

Order Plecoptera is the earliest, most diverse group of hemimetabolous insects, comprising more than 4000 described species under 17 families [8]. Their larvae live in cold lakes, pools, clean rocky creeks, rivers, and grassy places. The freshwater habitats and sandy regions are ideal for their larval development because the larval stages are highly susceptible to water pollution. The presence of stoneflies can be considered as a good indicator of water quality [9,10,11]. The genus *Isocapnia* [12] belongs to the Capniidae family and is predominantly distributed in North America and Asia [13,14]. The males of the genus *Isocapnia* often have an elongated epiproct, an apex with a distinctive form and a dark ventral vesicle extending from the sternum 9. Moreover, the female *Isocapnia* typically exhibits diagnostic sclerotization patterns on its subgenital plate [15]. To date, only 21 species of the genus *Isocapnia* have been described based on a morphological basis [8], but the molecular studies on this genus remain neglected; however, the current research provides initial molecular insights into the mitogenome features and phylogenetic placement. Currently, the most conventional classification of stoneflies was suggested by Zwick [16] using morphological research. In this study, Capniidae and four other families (Taeniopterygidae, Nemouridae, Notonemouridae, and Leuctridae) were grouped into Nemouroidea. The research demonstrated that Leuctridae and Capniidae were sister groups, which were named Leuctroidea, but the molecular studies did not confirm the monophyletic group Leuctroidea. Ding et al. [17] found that the Taeniopterygidae and Capniidae are sister groups, and Leuctridae was related to the rest of the Nemouroidea. In addition, Davis [18] also placed Taeniopterygidae and Capniidae as sister groups based on transcriptomic analysis, and several studies agreed with the research findings of Davis [19,20,21,22,23,24]. The initiation of next-generation sequencing techniques has led to a tremendous increase in the understanding of mitogenome studies; nonetheless, the mitogenome of Capniidae is still less documented. These limited data hinder accurate phylogenetic analysis [25,26]. More than 80 stoneflies have mitogenomes that are complete or almost complete, and these are now accessible in GenBank; however, only five mitogenomes of the Capniidae family were available [27,28]. While numerous phylogeny studies have been undertaken to identify the phylogenetic affinities amongst stoneflies, many of these affinities are still unclear. Nevertheless, inadequate taxon sampling renders these studies unsuitable for resolving exact phylogenetic associations within the Nemouroidea. The current study analyzed the first mitogenome of *Isocapnia anguis*, which will help to identify the genus classification as well as provide significant support for the evolutionary analysis of Nemouroidea. In addition, the composition of nucleotides, tRNAs secondary structure, and codon usage were also analyzed. Finally, a phylogenetic study of the superfamily Nemouroidea was undertaken using thirteen PCGs from accessible genomes. Hence, the current study aims to better understand the evolution of the Nemouroidea superfamily and the generic classification of the Capniidae family.

## 2. Materials and Methods

### 2.1. Sampling Collection and DNA Extraction

The specimens of *Isocapnia anguis* (Figure S1) [14] were collected from the China Sichuan Province (32°9.27′ N 104°1.44′ E, 2441 m) in 2016 and preserved in 75% ethanol. The terminalia was examined by KEYENCE VHX−5000 digital microscope and the final images were prepared using Adobe Photoshop CS6. The specimens were placed in the Insect collection of Yangzhou University (ICYZU), Jiangsu Province, China. This study was carried out without causing harm to endangered or threatened species and all scientific investigations were permitted. All specimens were kept in 100% ethanol at −20 °C. The genomic DNA was pulled out from the thoracic muscles and legs of adults using the column mtDNAoutKit (Axygen Biotechnology, Hangzhou, China), as endorsed by the supplier, and stored at −20 °C until used for PCR.

### 2.2. PCR Amplification and Sequencing

Mitogenome was amplified using LA-PCR and clear PCR implication under the following conditions: achieve initial denaturation at 93 °C for 02 min, then perform 40 cycles at 92 °C for 10 s; soften at 54 °C for 30 s; and extend at 68 °C for 8 min. The elongation rate was increased by 20 s for each cycle in the last 20 cycles, resulting in a final extension of 10 min at 68 °C. High throughput sequencing and universal primers were used to amplify mitochondrial genes in long overlapping fragments. The Axygen DNA gel Extraction Kit (Axygen Biotechnology, Hangzhou, China) was utilized to purify PCR results [19]. When the DNA had been extracted and refined, it was subjected to quality control, which involved testing via qubit 3.0 as well as electrophoresis on a 1% agarose gel. The NEBNext Ultra DNA Library Prep Kit for sequence analysis was used to generate a 500 bp matched library from high-quality DNA samples, and sequencing was performed on the Illumina NovaSeq 6000 platform (BIOZERON Co., Ltd., Shanghai, China). The Contigs of the mitogenome were generated by de novo assembly using GetOrganelle v1.6.5 as a reference for the mitochondrial genomes of closely related species. The potential mitochondrial readings were isolated from Illumina reads using BLAST against the mitogenomes species and GetOrganelle outcome. The mitochondrial Illumina states were achieved to complete mitogenome de novo assembly utilizing the SPAdes-3.13.1 program. The GetOrganelle contig was enhanced by the scaffolds from the SPAdes-3.13.1 program. To complete the process, the accumulated sequences were rearranged and orientated to match the reference mitochondrial genome, providing a complete genetic sequence of mitochondria (BIOZERON Co., Ltd., Shanghai, China).

### 2.3. Mitogenome Assembly and Annotation

The mitogenome assembly was performed using CodonCode Aligner (accessed on 5 February 2023). The mitogenomes from other species of order Plecoptera were used to recognize rRNAs, tRNAs, and PCGs genes, and ORFs were enclosed through ORF finder (accessed on 5 February 2022). The CGview tool server was used to create a circular map of the mitogenome [29]. We used the online MITOS program [30] to characterize the genes, with the default settings being used to predict PCGs, tRNA, and RNA (rRNA) genes. The structure of tRNA was predicted by the ARWEN algorithm using its default settings [31]. The codon usage, RSCU (relative synonymous codon usage) and nucleotide compositions were examined via MEGA V. 7.0 [32]. The AT-skew = (A − T)/(A + T) and the GC-skew = (G − C)/(G + C) formulas [33] were used to evaluate the skew of AT and GC data, respectively. The DNAMAN program was used to predict stem-loop structures in the CR, and tandem repeats of CR were assessed using the Tandem repeats finder online [34]. Mitogenome sequences of *I. anguis* were deposited in the GenBank as OQ735414 (Table 1).

### 2.4. Phylogenetic Analysis

We used thirty-one published Plecoptera and one newly sequenced mitogenome for the phylogenetic assessment, and two species of the family Perlodidae were treated as an outgroup (Table 1). The thirteen PCGs in thirty-two mitogenomes were synthesized using SequenceMatrix v. 1.7.8 [46] and aligned with the MAFFT algorithm [47], excluding stop codon. Before creating phylogenetic trees, the DAMBE v. 5.2 was used to check for nucleotide saturation. The MEGA V. 7.0 [32] was used to choose the GTR+G+I model as the optimal nucleotide substitution analytical model. The MrBayes (version 3.1.2) was used to conduct a Bayesian inference (BI) study under the following conditions: 10 million generations with 100-generation intervals, 4 chains, and a burn-in of 25% of the tree [23,48,49]. The maximum likelihood (ML) was conducted using IQTree v. 1.6.12 [50] with 1000 ultrafast bootstrap estimates, and the tree was visualized with Fig Tree v. 1.4.2.

## 3. Results and Discussion

### 3.1. Characteristics of the Mitogenome

**Figure 1:**
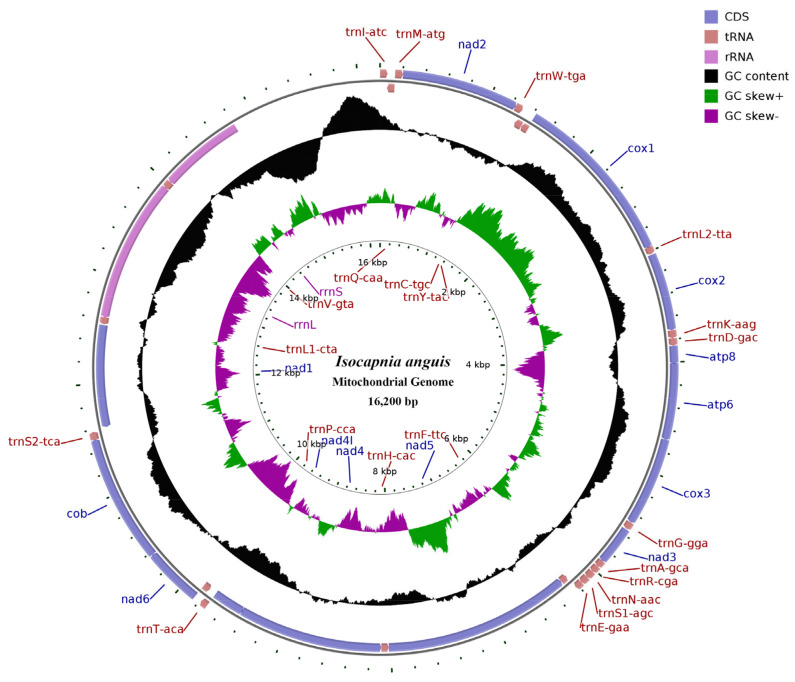
Map of the mitogenome of *I. anguis*. Genes outside the map are recorded clockwise, while genes inside the map are counterclockwise. The interior spheres showed GC content (Guanine and Cytosine nucleotides) and the GC-skew, plotted as the deviation from of overall mean value of the whole sequence.

The length of the whole mitogenome of *I. anguis* is 16,200 base pairs (bp) (Figure 1), which is virtually identical to the genome of *Capnia zijinshana* (16,310 bp) and comparable to prior researches attempting to study the entire mitogenomes of stoneflies (Table 1). Among the Capniidae complete mitogenomes, the length variation was minimal in PCGs, tRNAs, and rRNAs, but differences in the control region are high. The mitogenome is a circular DNA; its components include 13 PCGs, 22 tRNAs, 2 rRNA genes, and a control region, also recognized as a non-coding region (Figure 1, Table 2). Twenty-three genes (9 PCGs and 14 tRNAs) were detected on the J−strand (major strand), the remaining genes (4 PCGs, 8 tRNAs, and 2 rRNAs) being oriented on the N−strand (minor strand) (Table 2, Figure 1). The gene order of *I. anguis* is identical to other sequenced stoneflies and to *Drosophila yakuba*, which is reflected in the hypothesized ancestral mitogenome [51,52]. In *I. anguis*, 53 overlapping nucleotides were positioned in 15 pairs of adjacent genes, ranging from 1 to 8 bp; the most extended overlap (8 bp) was found among *trnTrp (W)* and *trnCys (C)*. Only 41 IGN (intergenic nucleotides) were observed in eight various locations, and their lengths ranged from 1 to 16 base pairs (bp), with the longest IGN (16 bp) located amongst *trnSer2 (UCN)* and *nad1*.

### 3.2. Base Composition

In *I. anguis*, the overall nucleotide composition is 31.28% T, 20.64% C, 34.91% A, and 13.17% G, correspondingly (Table 2). The A + T content of the entire mitogenome, PCGs, PCG−J, PCG−N, rRNAs, tRNAs, and the non-coding region is 66.19%, 64.78%, 62.95, 66.52%, 71.20%, 69.02% and 66.62%, respectively (Table 2). The A + T content is 86.15% for *trnGlu (E)*, and the lowest was 56.25% for *trnArg (R)* (Table S1). The A + T content of mitogenome is identical with *Apteroperla tikumana* (66.51%), while a slightly increase was seen in *C. zijinshana* (68.5%) compared to *I. anguis* species. The bias in the nucleotide makeup was assessed by AT- and GC-skew, as previously described [33]. Typically, the nucleotide contents of metazoan mitogenomes have a distinct strand bias [53,54].

In this study, the *I. anguis* whole mitogenome exhibited a positive AT-skew (0.05) and negative GC-skew (−0.22), while the PCGs and PCG−J (PCGs encoded by the majority strand) both showed a negative AT- and GC-skew and PCG−N (PCGs encoded by the minority strand) showed positive GC-skew and negative AT-skew (Table 2). For the J−strand, the mitogenomes for most insects showed a positive AT-skew and negative GC-skew [55], while in this study, PCG and PCG−J both showed negative AT- and GC- skew. The A + T bias in *I. anguis* was linked to asymmetric mutation and selection pressure during transcription and replication, which was demonstrated to be functional in mitogenome studies [33,34,56].

### 3.3. Protein Coding Genes

The full size of the thirteen PCGs of *I. anguis* was 11,219 bp, and its A + T content stood at 64.78% (Table 2). In the *I. anguis* mitogenome, twelve PCGs initiated with the conventional start codon ATN (ATG, ATA or ATT), whereas *nad5* gene began with codon GTG. Eleven PCGs had TAN (TAA or TAG) as their last codon; however, *cox1* and *nad5* both had shortened termination codons and concluded with T (Table S1). The use of incomplete stop codon T is mutual in most of the stoneflies [20,23,27,28,40,57] and other animals’ mitogenome [5], forming a comprehensive TAA terminal signal via post-transcriptional polyadenylation [58,59].

**Figure 2:**
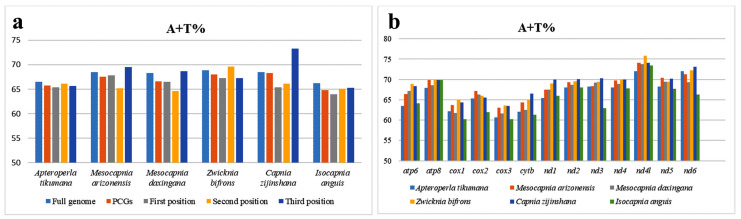
Comparison of nucleotide components of mitogenome across the Capniidae family: (**a**) A + T% of different positions; (**b**) A + T% of PCGs.

**Figure 3:**
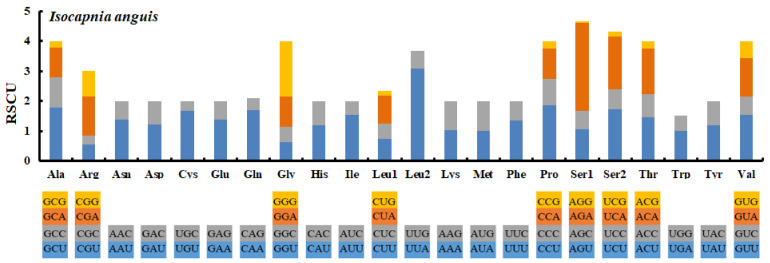
The relative synonymous codon usage (RSCU) in the *I. anguis* mitogenome.

In the entire mitogenome, the A + T content ranged from 65.19% to 68.89% in the Capniidae species (Figure 2, Table S2). Comparison among the PCGs (Figure 2, Table S3) of all Capniidae showed that the A + T content of *cox3* was the lowest (62.10%), followed by *cox1* (62.83%) and *cytb* (63.63%), whereas *nd4l, nd6*, and *atp8* were the highest (73.85%, 70.69%, and 69.285%), respectively (Figure 2, Table S3). Furthermore, PCGs have a substantially greater A + T concentration in the third codon position (ranging from 65.27% to 73.27%) than in the first and second (ranging from 65.08% to 69.57%) (Figure S1). We calculated the RSCU values of the *I. anguis* (Figure 3). The GCT (Ala), TGT (Cys), CAA (Gln), ATT (Ile), TTA (Leu), CCT (Pro), and TCT (Ser) were comparatively high, whereas UUG (Leu1) and CCG (Pro) were used the least (Figure 3). This differs from other species of stoneflies [19,28], so we believe that perhaps this was unique to the genus *Isocapnia*. Nevertheless, further research and data are necessary to provide confirmation.

### 3.4. Transfer RNA Genes

**Figure 4:**
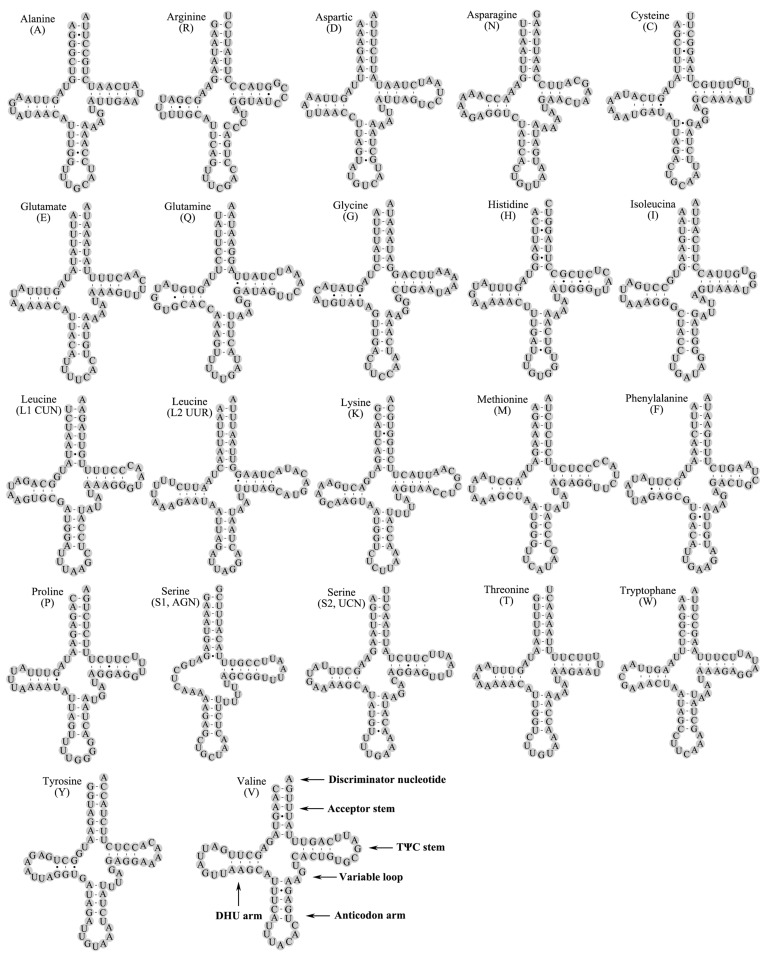
Secondary structures of 22 tRNAs of *I. anguis*. All tRNAs are labeled with the abbreviations of their consistent amino acids. Dashes (-) specify Watson–Crick base combination and dots (•) specify G-U base pairing.

The *I. anguis* mitogenome had 22 tRNA genes scattered all over the PCGs and rRNAs. The 22 tRNAs were 1475 bp with a length ranging from 64 to 71 bp and had an A + T content of 69.02% (Table 2). Most tRNAs had a distinctive cloverleaf structure (Figure 4), with the exception of *trnSer (AGN)*, which lacked the dihydrouridine (DHU) arm and formed a small loop, as is usually the case in mammals and in numerous metazoans [60]. The anticodon of all tRNAs was identical to that of stoneflies. A total of 41 miss-matched base pairs were detected in the tRNAs, which are 2 bp U−U, 5 bp A−U, 2 bp U−C, 1 bp A−C, 1 bp A−G, and 30 bp weak G−U pairs (Figure 4), which produced weak bonds in tRNAs and non-canonical pairs in the secondary structure of tRNAs [61]. The well-maintained and variable position in tRNA structures were detected in the TΨC loop and anticodon arm, respectively (Figure 4).

There were 2 rRNAs in the *I. anguis* mitogenome with a total size of 2125 bp and an A + T content of 71.20% (Table 2). The boundaries of the small and large subunit ribosomal RNAs (*srRNA* and *lrRNA*) were found using previously published plecopteran sequence alignment. The large rRNA subunit gene (*lrRNA*) is 1333 bp long, has an A + T content of 72.47%, and was found between *trnLeu1 (CUN)* and *trnVal (V).* The small rRNA gene (*srRNA*) was 792 bp long and had a 69.07% A + T content, which is located between *trnVal (V)* and the non-coding region (Table S1). Both *lrRNA* and *srRNA* size and characteristics are consistent with those reported in well-documented Plecoptera species [19,20,21,22,23,24,25,26,27,28].

### 3.5. The Control Region

The control region is the most variable part of the mitogenome due to the insertion and deletion measures, deviation in domain length fluctuation, and the number of tandem repeats [62,63], which contain vital components for the initiation of transcription and replication [64]. The CR of the *I. anguis* mitogenome was 1392 bp with an A + T content of 66.6%, which was located amongst *rrnS* and *trnlle* (Table S1). The length of the CR in Capniidae mitogenomes was more prolonged compared to other stoneflies with a lower A + T content. The CR of *C. zijinshana* was substantially longer among Capniidae mitogenomes 1513 bp in length, with a lower A + T content of 62.0% [28].

**Figure 5:**
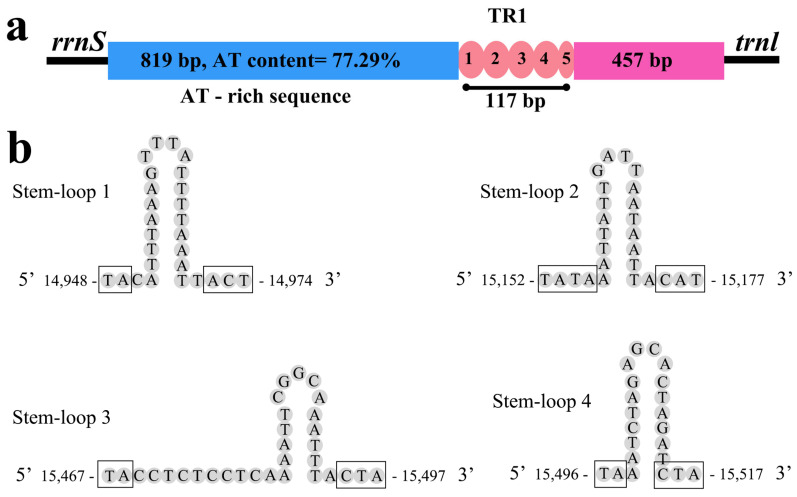
Control region of *I. anguis* mitogenome. (**a**) Structure elements of CR. (**b**) Stem-loop structures in the CR of *I. anguis*. The two-sided nucleotide motifs of stem-loop (TA)n, CAT, C(T)nA are specified by squares.

The CR of the *I. anguis* can be divided into three parts; (1) a leading sequence 819 bp adjacent to *srRNA* with high A + T content; (2) one tandem repeat (TR) sequence blocks consisting of repeats units; (3) the rest of the control region (457 bp) (Figure 5). The tandem repeats are located between 15,626–15,742 bp and four stem-loop structures (14,948–14,974 bp, 15,152–15,177 bp, 15,467–15,497 bp, 15,496–15517 bp) (Figure 5). The SL structure was a single root with (TA)n structures on the left side and CAT or C (T)nA structures on the right side (Figure 5). The study suggested that these SL structures and CR tandem repeats may have stimulatory effects on mitogenome replication and transcription [65,66].

### 3.6. Phylogenetic Analysis

**Figure 6:**
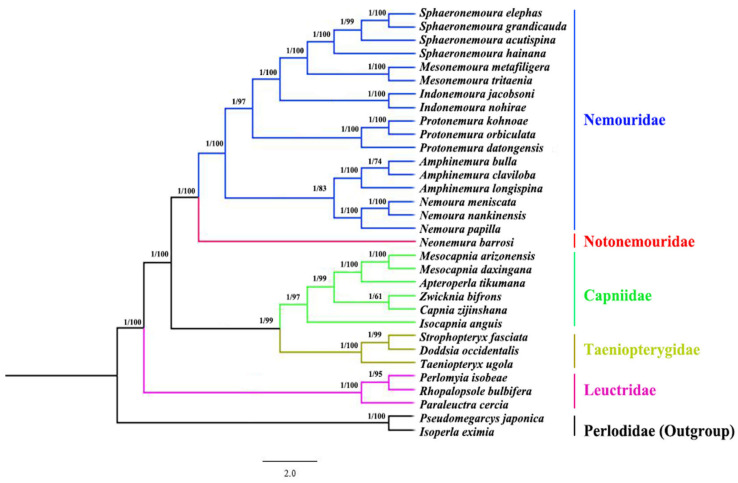
Phylogenetic tree of superfamily Nemouroidea based on the mitogenomes of 32 stoneflies using BI (Bayesian inference) and ML (Maximum Likelihood). Numbers at nodes represent posterior probabilities (**left**) and bootstrap values (**right**). Families are written to the right side of each species. *Pseudomegarcys japonica*, *Isoperla eximia* served as outgroup species.

The phylogenetic analysis of the whole Nemouroidea was constructed using thirteen PCGs from thirty-two Plecoptera species. These mitogenomes included seventeen species from the family Nemouridae, one species of the Notonemouridae, six species from the Capniidae, three species each from Taeniopterygidae and Leuctridae, while two species of Perlodidae (*Isoperla eximia* and *Pseudomegarcys japonica*) were included as outgroups (Table 1). The phylogeny tree patterns were analogous to dendrograms derived from BI and ML analysis. The topological tree structures were similar for both ML and BI analysis. The monophyly of every group was highly supported (Post probability values (PP) = 1.00 and bootstrap values (BP) > 99). This study represented the phylogeny of the superfamily Nemouroidea, which includes five families, such as Nemouridae, Notonemouridae, Capniidae, Taeniopterygidae, and Leuctridae. Our analysis substantially supported Leuctridae + ((Capniidae + Taeniopterygidae) + (Nemouridae +Notonemouridae)). Moreover, the Leuctridae family was the initial branch to be developed within the Nemouroidea superfamily. It was determined in our study that Nemouridae is the sister group of Notonemouridae (BP = 100, PP = 1.00) (Figure 6). These results correlate with the morphological hypothesis [16] and mitogenome studies of [20,21,22,23,24], but few molecular analyses disagree with this placement [67,68]. According to the studies of Zwick [16], the Capniidae family was initially positioned as a sister to the Leuctridae. After this, the Capniidae was clustered with the Nemouridae and Notonemouridae. The current results supported Capniidae as a sister taxon to Taeniopterygidae (BP = 98, PP = 1.00) (Figure 6). This hypothesis varies from the morphological hypothesis that Leuctridae and Capniidae are sister taxon [16], but until now, no molecular study has supported this placement. In previous mitogenome studies [17,19,20,21,22,23,24], the Capniidae and Taeniopterygidae were considered as sister groups, and Leuctridae to the remaining Nemouroidea. However, the results of this study demonstrated more stability (BP =99, PP = 1.00) (Figure 6) than prior studies [17,24].

In the generic level of the Capniidae family, the genus *Isocapnia* is a sister to *Capnia* (BP = 98, PP = 1.00) (Figure 6). The monophyly of each genus is often strongly supported (BP > 97 and PP > 0.99), except for *Zwicknia* and *Capnia* genera. *Zwcknia* and *Capnia* were more closely related, yet their bootstrap probability values are lower than those of other genera. *Zwicknia* is a newly erected genus from the *Capnia bifrons* species group [69], and each genus has just one species sequenced so far. As data for only a few taxa of the Capniidae family are currently available, additional molecular testing from a wider variety of genera and an increased number of species are required to better resolve the phylogeny of this group. Finally, in this study, the best well-supported generic phylogenetic relationship within Capniidae is as follows: (*Isocapnia* + (*Capnia* + *Zwicknia*) + (*Apteroperla* + *Mesocapnia*)). However, to form a better understanding, more molecular investigations are needed to resolve the phylogeny of the Capniidae family.

## 4. Conclusions

The contemporary phylogeny of Capniidae is based on traditional classification and is universally recognized. However, molecular evidence supports different hypotheses. Since only five mitogenomes are currently available, more research data on other genera and species are required to determine the precise phylogenetic affiliation of this family. In this study, the first mitogenome of the genus *Isocapnia* was sequenced. The *Isocapnia* mitogenome was found to be highly conserved in terms of size, gene order, and nucleotide sequence when compared to other members of the Capniidae family. Both the ML and BI approaches were used to infer phylogenetic trees, and these trees created identical topologies across all 13 PCGs. The best well-supported generic phylogenetic relationship within Capniidae is as follows: (*Isocapnia* + (*Capnia* + *Zwicknia*) + (*Apteroperla + Mesocapnia*)). The relationship of the superfamily Nemouroidea recovered as strongly supported Leuctridae + ((Capniidae + Taeniopterygidae) + (Nemouridae + Notonemouridae)). These findings will help us to understand the general classification and mitogenome structure of the Capniidae family and their relationship within Nemouroidea.

## Figures and Tables

**Table 1:** Common information of Nemouroidea species analyzed in this study.

Family	Species	Number (bp)	GenBank Number	References
Nemouridae	*Sphaeronemoura grandicauda*	15,661	MH085454	[20]
	*Sphaeronemoura acutispina*	15,016	MH085455 *	[35]
	*Sphaeronemoura hainana*	15,260	MK111420 *	[17]
	*Sphaeronemoura elephas*	15,846	MN944385	[36]
	*Mesonemoura tritaenia*	15,778	MH085451	[37]
	*Mesonemoura metafiligera*	15,739	MH085450	[37]
	*Protonemura kohnoae*	15,707	MH085452	[20]
	*Protonemura datongensis*	15,756	MT276842	[38]
	*Protonemura orbiculata*	15,758	MH085453	[20]
	*Amphinemura claviloba*	15,707	MN720741	[39]
	*Amphinemura bulla*	15,827	MW339348	[40]
	*Amphinemura longispina*	15,709	MH085446	[20]
	*Indonemoura nohirae*	15,738	MH085449	[20]
	*Indonemoura jacobsoni*	15,642	MH085448	[20]
	*Nemoura papilla*	15,774	MK290826	[41]
	*Nemoura nankinensis*	16,602	KY940360	[42]
	*Nemoura meniscata*	15,895	MN944386	[43]
Notonemouridae	*Neonemura barrosi*	14,852	MK111418 *	[17]
Capniidae	*Mesocapnia daxingana*	15,524	KY568983 *	[27]
	*Mesocapnia arizonensis*	14,921	KP642637 *	[44]
	*Capnia zijinshana*	16,310	KX094942	[28]
	*Apteroperla tikumana*	15,564	NC_027698	[35]
	*Zwicknia bifrons*	15,380	MT872688*	[35]
	*Isocapnia anguis*	16,200	OQ735414	This study
Taeniopterygidae	*Doddsia occidentalis*	16,020	MG589787	[19]
	*Strophopteryx fasciata*	15,527	ON500674	[24]
	*Taeniopteryx ugola*	15,353	MG589786	[19]
Leuctridae	*Paraleuctra cercia*	15,625	MK492251	[21]
	*Rhopalopsole bulbifera*	15,599	MK111419 *	[17]
	*Perlomyia isobeae*	15,795	MK492252	[21]
Perlodidae(Outgroup)	*Isoperla eximia*	16,034	MG910457	[45]
	*Pseudomegarcys japonica*	16,067	MG910458	[45]

* Incomplete mitogenome sequence.

**Table 2:** The nucleotide components of the *I. anguis* mitogenome.

Feature	Proportion of Nucleotides								No. of Nucleotides
	T%	C%	A%	G%	AT%	GC%	AT Skew	GC Skew	
Genome	31.28	20.64	34.91	13.17	66.19	33.81	0.05	−0.22	16,200
Protein coding genes	37.63	18.62	27.15	16.60	64.78	35.22	−0.16	−0.06	11,219
First position	36.58	18.61	27.38	17.41	63.96	36.02	−0.14	−0.03	3739
Second position	37.94	18.00	27.14	16.90	65.08	34.90	−0.17	0.03	3739
Third position	38.37	19.25	26.90	15.46	65.27	34.71	−0.18	−0.11	3739
PCG−J	34.34	22.55	28.61	14.50	62.95	37.05	−0.09	−0.22	6904
First codon position	28.67	22.42	34.01	14.90	62.68	37.32	0.09	−0.20	2302
Second codon position	35.16	22.56	24.12	18.17	59.28	40.72	−0.19	−0.11	2301
Third codon position	39.20	22.69	27.68	10.43	66.88	33.12	−0.17	−0.37	2301
PCG−N	40.88	14.82	25.64	18.67	66.52	33.48	−0.23	0.12	5609
First codon position	40.91	12.62	26.68	19.79	67.59	32.41	−0.21	0.22	1870
Second codon position	40.77	17.71	20.65	20.87	61.42	38.58	−0.33	0.08	1869
Third codon position	40.96	14.12	29.57	15.35	70.53	29.47	−0.16	0.04	1870
tRNA genes	34.24	13.69	34.78	17.29	69.02	30.98	0.01	0.12	1475
rRNA genes	37.51	10.45	33.69	18.35	71.20	28.80	−0.05	0.27	2125
lrRNA	37.66	9.08	34.81	18.45	72.47	27.53	−0.04	0.34	1333
srRNA	37.25	12.75	31.82	18.18	69.07	30.93	−0.08	0.18	792
CR	30.08	20.67	36.54	12.71	66.62	33.38	0.10	−0.24	1393

## Data Availability

Data are available on reasonable request.

## Supplementary Materials

The following supporting information can be downloaded at: https://www.mdpi.com/article/10.3390/genes14050965/s1, Table S1: Organization of the *I. anguis* mitochondrial genome; Table S2: A + T content of different components or positions among mitogenome of the family Capniidae; Table S3. A + T content of different PCGs among mitogenome of the family Capniidae; Figure S1. (A) *I. anguis* adult habitus dorsal view, (B) head and thorax dorsal view, (C) terminalia dorsal view.
