# Supplementary material for: The First Complete Mitochondrial Genome of Genus Isocapnia (Plecoptera: Capniidae) and Phylogenetic Assignment of Superfamily Nemouroidea
Source: Genes (Basel). 2023 Apr 24;14(5):965. doi: 10.3390/genes14050965 (PMC10217884; doi:10.3390/genes14050965)
Supplement: Supplementary file 1 [file genes-14-00965-s001.zip › genes-2285305-supplementary.pdf]

Table S1. Organization of the *I. anguis* mitochondrial genome

Gene	Position (bp)	Size (bp)	Direction	Intergenic Nucleotides (IGN)	Anti- or Start/ Stop Codons	A+T%
trnIle (I)	F	1-66	66	-	GAT	62.12
trnGln (Q)	R	64-132	69	-3	TTG	69.57
trnMet (M)	F	133-201	69	0	CAT	62.32
nad2	F	202-1236	1035	0	ATG/TAA	68.02
trnTrp (W)	F	1235-1302	68	-2	TCA	70.59
trnCys (C)	R	1295-1362	68	-8	GCA	69.12
trnTyr (Y)	R	1365-1430	66	2	GTA	62.12
cox1	F	1423-2967	1545	-8	ATT/TAA	60.26
trnLeu2 (UUR)	F	2963-3028	66	-5	TAA	65.15
cox2	F	3035-3722	688	6	ATG/T	61.92
trnLys (K)	F	3723-3792	70	0	CTT	62.86
trnAsp (D)	F	3793-3859	67	0	GTC	79.10
atp8	F	3860-4018	159	0	ATT/TAA	69.81
atp6	F	4012-4689	678	-7	ATG/TAA	64.16
cox3	F	4689-5477	789	-1	ATG/TAA	60.20
trnGly (G)	F	5479-5545	67	1	TCC	73.13
nad3	F	5552-5899	348	6	ATA/TAG	62.93
trnAla (A)	F	5898-5961	64	-2	TGC	67.19
trnArg (R)	F	5962-6025	64	0	TCG	56.25
trnAsn (N)	F	6026-6092	67	0	GTT	73.13
trnSer1 (AGN)	F	6093-6160	68	0	GCT	64.71
trnGlu (E)	F	6161-6225	65	0	TTC	86.15
trnPhe (F)	R	6224-6288	65	-2	GAA	67.69
nad5	R	6289-8020	1732	0	GTG/T	67.67
trnHis (H)	R	8021-8088	68	0	GTG	64.71
nad4	R	8089-9429	1341	0	ATG/TAG	67.79
nad4l	R	9423-9719	297	-7	ATG/TAA	73.40
trnThr (T)	F	9722-9786	65	2	TGT	81.54
trnPro (P)	R	9787-9852	66	0	TGG	69.70
nad6	F	9854-10378	525	1	ATT/TAA	66.29
Cytb	F	10378-11514	1137	-1	ATG/TAG	61.30
trnSer2 (UCN)	F	11513-11582	70	-2	TGA	74.29
nad1	R	11599-12543	945	16	ATA/TAG	65.93
trnLeu1 (CUN)	R	12551-12616	66	7	TAG	71.21
rrnL	R	12616-13948	1333	-1	-	72.47
trnVal (V)	R	13946-14016	71	-3	TAC	66.20
rrnS	R	14016-14807	792	-1	-	69.07
CR		14808-16200		0	-	66.62

Table S2. A + T content of different components or positions among mitogenome of the family Capniidae

Species Name	Full genome	PCGs	First position	Second position	Third position
Apteroperla tikumana	66.51	65.73	65.42	66.11	65.68
Mesocapnia arizonensis	68.49	67.54	67.85	65.23	69.54
Mesocapnia daxingana	68.34	66.57	66.46	64.59	68.65
Zwicknia bifrons	68.89	68.03	67.22	69.57	67.29
Capnia zijinshana	68.47	68.27	65.41	66.17	73.24
Isocapnia anguis	66.19	64.78	63.96	65.08	65.27

Table S3. A + T content of different PCGs among mitogenome of the family Capniidae

Species Name	*atp6*	*atp8*	*cox1*	*cox2*	*cox3*	*cytb*	*nd1*	*nd2*	*nd3*	*nd4*	*nd4l*	*nd5*	*nd6*
Apteroperla tikumana	63.42	67.92	62.14	65.26	60.71	62.08	65.46	68.02	68.22	68.01	72.03	68.21	72
Mesocapnia arizonensis	66.37	69.81	63.7	67.15	62.99	64.38	67.51	69.28	68.36	69.72	74.07	70.38	71.24
Mesocapnia daxingana	67.11	68.55	61.69	66.28	61.6	62.53	67.51	68.7	69.21	68.83	73.74	69.4	69.33
Zwicknia bifrons	68.88	69.81	64.94	65.99	63.62	65.00	68.98	69.57	69.39	69.95	75.82	69.4	72.19
Capnia zijinshana	68.29	69.81	64.3	65.55	63.5	66.49	69.93	70.02	70.34	69.95	74.07	70.21	73.14
Isocapnia anguis	64.16	69.81	60.26	61.92	60.2	61.3	65.93	68.02	62.93	67.79	73.4	67.67	66.29
Average	66.37	69.28	62.83	65.35	62.10	63.63	67.55	68.93	68.07	69.04	73.85	69.21	70.69

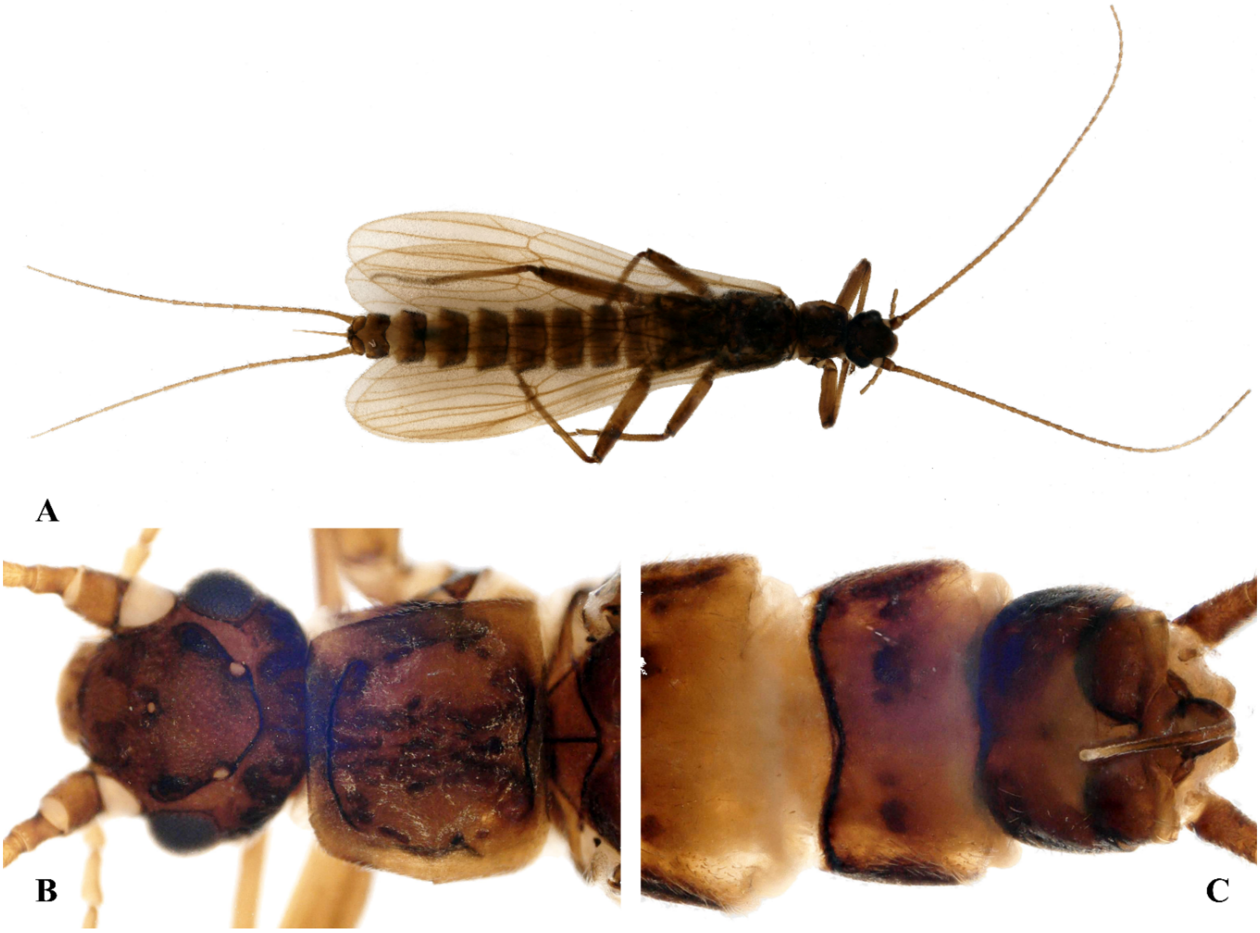

Figure S1. (A) *I. anguis* adult habitus dorsal view, **(B)** head and thorax dorsal view, **(C)** terminalia dorsal view.
